# Chinese medicine in the treatment of non-alcoholic fatty liver disease based on network pharmacology: a review

**DOI:** 10.3389/fphar.2024.1381712

**Published:** 2024-04-17

**Authors:** Shihao Zheng, Chengyuan Xue, Size Li, Xiaobin Zao, Xiaoke Li, Qiyao Liu, Xu Cao, Wei Wang, Wenying Qi, Peng Zhang, Yongan Ye

**Affiliations:** ^1^ Dongzhimen Hospital, Beijing University of Chinese Medicine, Beijing, China; ^2^ Beijing University of Chinese Medicine, Beijing, China; ^3^ Key Laboratory of Chinese Internal Medicine of Ministry of Education and Beijing, Dongzhimen Hospital, Beijing University of Chinese Medicine, Beijing, China; ^4^ Liver Diseases Academy of Traditional Chinese Medicine, Beijing University of Chinese Medicine, Beijing, China; ^5^ Dongfang Hospital, Beijing University of Chinese Medicine, Beijing, China

**Keywords:** traditional Chinese medicine, non-alcoholic fatty liver disease, network pharmacology, research progress, review

## Abstract

Non-alcoholic fatty liver disease (NAFLD) is a clinicopathological syndrome characterized by abnormalities in hepatic fat deposition, the incidence of which has been increasing year by year in recent years. It has become the largest chronic liver disease globally and one of the important causes of cirrhosis and even primary liver cancer formation. The pathogenesis of NAFLD has not yet been fully clarified. Modern medicine lacks targeted clinical treatment protocols for NAFLD, and most drugs lack efficacy and have high side effects. In contrast, Traditional Chinese Medicine (TCM) has significant advantages in the treatment and prevention of NAFLD, which have been widely recognized by scholars around the world. In recent years, through the establishment of a “medicine-disease-target-pathway” network relationship, network pharmacology can explore the molecular basis of the role of medicines in disease prevention and treatment from various perspectives, predicting the pharmacological mechanism of the corresponding medicines. This approach is compatible with the holistic view and treatment based on pattern differentiation of TCM and has been widely used in TCM research. In this paper, by searching relevant databases such as PubMed, Web of Science, and Embase, we reviewed and analyzed the relevant signaling pathways and specific mechanisms of action of single Chinese medicine, Chinese medicine combinations, and Chinese patent medicine for the treatment of NAFLD in recent years. These related studies fully demonstrated the therapeutic characteristics of TCM with multi-components, multi-targets, and multi-pathways, which provided strong support for the exact efficacy of TCM exerted in the clinic. In conclusion, we believe that network pharmacology is more in line with the TCM mindset of treating diseases, but with some limitations. In the future, we should eliminate the potential risks of false positives and false negatives, clarify the interconnectivity between components, targets, and diseases, and conduct deeper clinical or experimental studies.

## Introduction

**FIGURE 1:**
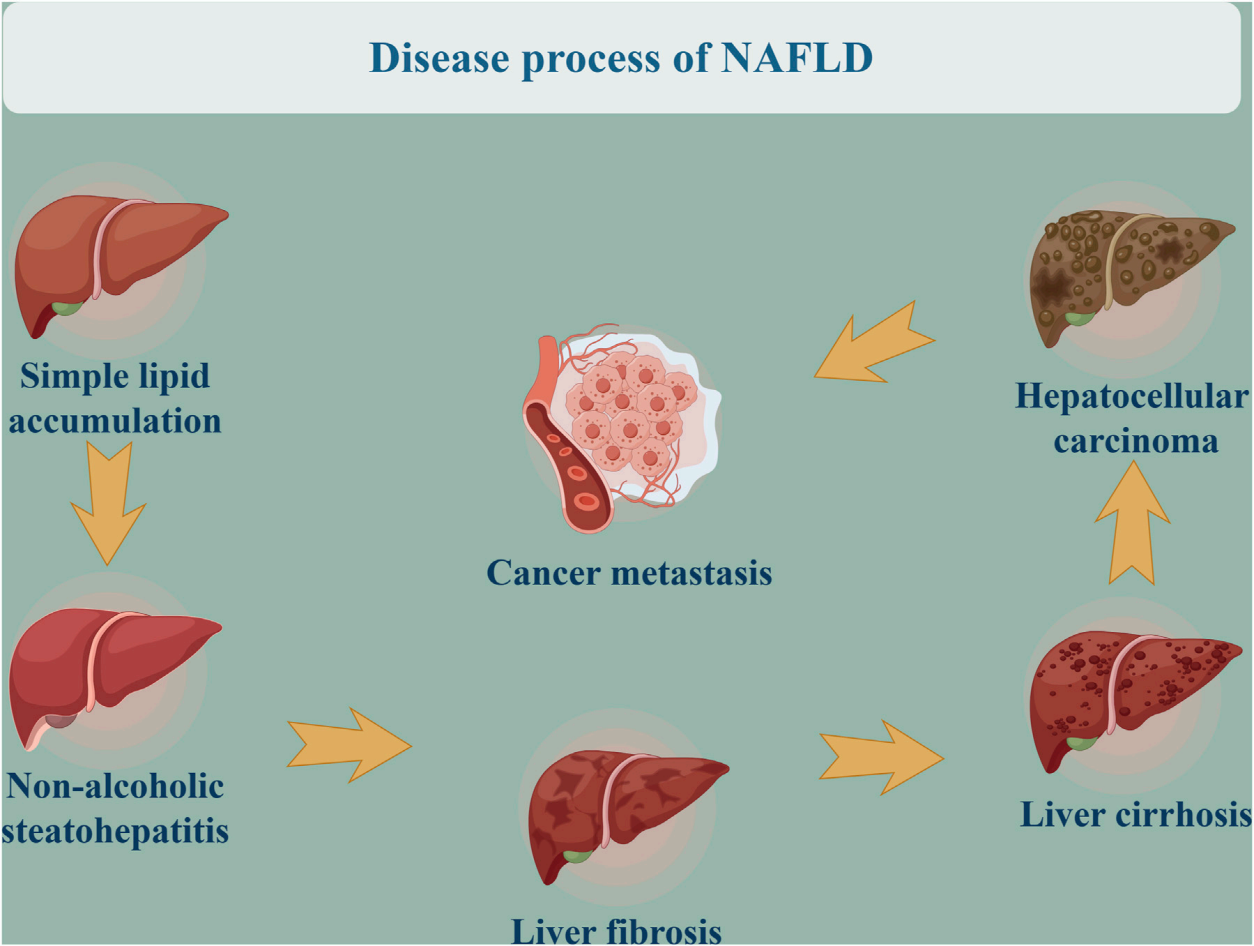
Disease process of NAFLD.

NAFLD is a clinicopathologic syndrome characterized by abnormal fat deposition in the liver, induced by factors other than alcohol, genetic diseases, and drugs (Powell, et al., 2021; Adams, et al., 2005). The incidence of NAFLD is increasing year by year, making it the world’s largest chronic liver disease. Studies have found that the prevalence rate of NAFLD is about 25.2% globally (Huang, et al., 2021), reaching as high as 29.6% in Asian countries (Li, et al., 2019a). In the disease process and pathological changes of NAFLD, as a continuous spectrum of diseases, the development of NAFLD encompasses different pathological stages. Simple lipid accumulation represents the early stage of NAFLD, wherein the fat content of the liver exceeds 5%, with or without mild inflammation. With the further aggravation of hepatocellular damage and liver inflammation, approximately 15%–25% of patients with simple fatty liver disease progress to non-alcoholic steatohepatitis (NASH). As shown in Figure 1, this progression may subsequently lead to liver fibrosis, liver cirrhosis, even hepatocellular carcinoma and cancer metastasis, posing a serious threat to patients’ lives (Sheka, et al., 2020; Pouwels, et al., 2022; Wang, et al., 2024). Relevant studies (Takayama, et al., 2021) have identified NAFLD as one of the most significant causes of liver cirrhosis and even primary liver cancer, with 37% of NAFLD patients developing liver cirrhosis. Contemporary research on the pathogenesis of NAFLD predominantly centers on the traditional “second strike” hypothesis and the more recent “multiple strike” hypothesis, which encompass factors such as lipid accumulation, insulin resistance, genetic predisposition, and inflammatory processes (Başaranoğlu and Örmeci, 2014). Despite the incomplete elucidation of NAFLD pathogenesis, a substantial body of literature suggests that early hepatic steatosis significantly contributes to the development of the disease (Rotman and Sanyal, 2017). Hepatic steatosis is the result of dysregulation in lipid uptake and disposal mechanisms, primarily involving pathways such as *de novo* lipogenesis, free fatty acid uptake, fatty acid beta-oxidation, and export via very low density lipoprotein from hepatocytes (Ipsen, et al., 2018). These lipid metabolic pathways are under the control of various proteins, and genetic variations in lipid metabolism-related protein genes can alter protein activity, disrupting one or more lipid metabolic pathways and ultimately leading to hepatic lipid accumulation and the development of NAFLD.

From a clinical perspective, the symptoms of NAFLD are typically non-specific, encompassing fatigue, dyspepsia, dull pain in the liver region, and metabolic abnormalities including overweight, visceral obesity, and lipid dysregulation. NAFLD represents a metabolic liver injury associated with insulin resistance and genetic predisposition. As the prevalence of obesity and diabetes continues to rise, the worldwide incidence of NAFLD is also escalating, posing a significant threat to public health. NAFLD has a significant impact on the hepatobiliary system of patients and is closely associated with insulin resistance, lipid disorders, atherosclerosis, fat embolism, and hematologic diseases, all of which contribute to a poor clinical prognosis (Friedman, et al., 2018). While lifestyle and dietary modifications can effectively ameliorate NAFLD, prolonged disease progression may necessitate pharmacological intervention for optimal management. Ongoing research into the pathogenesis of NAFLD is leading to the development of therapeutic drugs aimed at improving outcomes for patients with this condition. Presently, conventional pharmacological treatments for NAFLD encompass lipid-lowering agents, hepatoprotective anti-inflammatory medications, among others. Notably, on 15 March 2024, the U.S. Food and Drug Administration (FDA) announced the approval of the oral small molecule drug Rezdiffra (the active ingredient is resmetirom) for the treatment of NASH patients with liver fibrosis, along with diet and exercise together, which can effectively alleviate liver damage (Kokkorakis, et al., 2024; The Lancet Gastroenterology, 2024). Agonists of thyroid hormone receptor (THR)-β, which is highly expressed in human liver, is able to regulate lipid metabolism, lowering low-density cholesterol and triglyceride (TG), among others. In addition, THR-β reduces lipotoxicity and improves liver function, which in turn reduces the accumulation of liver fat. Therefore, THR-β agonists have the potential to modulate multiple hepatic metabolic pathways to treat NASH(Karim and Bansal, 2023; Tacke, et al., 2023). Rezdiffra, the first FDA-approved NASH drug to be marketed as THR-β in 40 years, reduces liver fat accumulation and relieves liver inflammation. However, Rezdiffra also has certain side effects, including nausea and diarrhea, which should also be evaluated for clinical use. In addition to this, in recent years, a wide range of scholars have explored peroxisome proliferator-activated receptors, genetic factors, glucagon-like peptides 1, agents influencing hepatic steatosis synthesis, and a range of other pathways, and some combination drug trials are already underway (Noureddin, 2024). Despite the initial establishment of the pathogenesis of NAFLD within contemporary medical understanding, there remains a dearth of tailored clinical intervention strategies, with many existing drugs exhibiting limited efficacy and notable side effects. From the perspective of TCM, the classical books of TCM do not explicitly mention the disease name “fatty liver.” However, based on the symptoms and progression of the disease, it aligns with the clinical presentation of hypochondriac pain or liver and spleen enlargement in patients with NAFLD in modern medicine. Many contemporary medical practitioners classify the disease under the categories of “Xie Tong” and “Gan Pi” based on its symptoms and clinical manifestations. The State Administration of TCM of China named NAFLD as “Gan Pi”, because it includes the evolution of the whole disease including simple fatty liver, steatohepatitis, related cirrhosis and hepatocellular carcinoma. In comparison to western medicine, TCM has demonstrated a distinct advantage in the management of NAFLD by effectively regulating lipid levels and enhancing liver function. Numerous TCM practitioners have successfully utilized traditional methods to treat NAFLD, yielding significant therapeutic outcomes and indicating a favorable outlook for the future.

**FIGURE 2:**
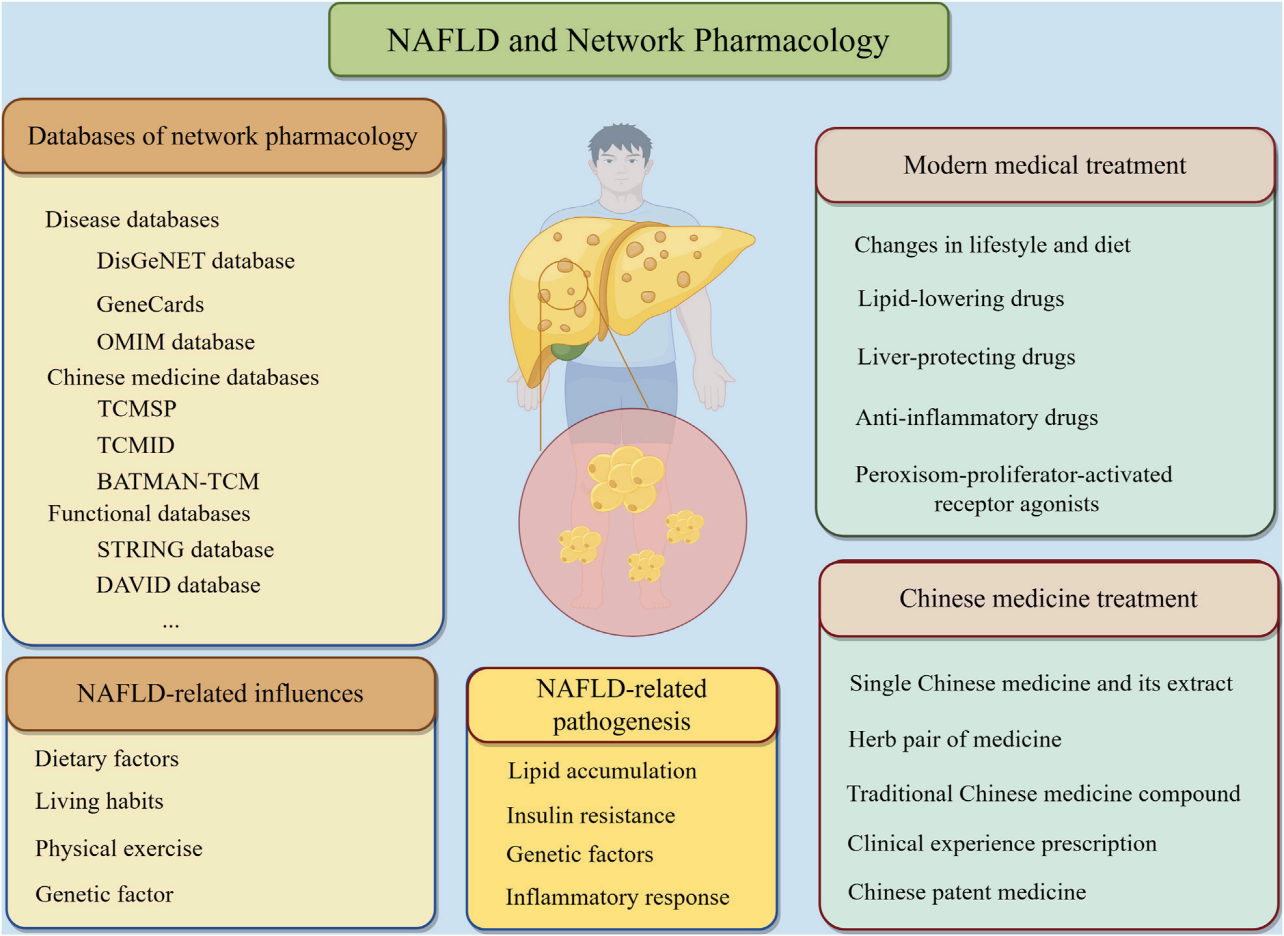
NAFLD and network pharmacology.

The term “network pharmacology” was first introduced by British pharmacologist Hopkins (Hopkins, 2007) in 2007, along with specific research concepts and methodologies. This discipline has gained widespread recognition globally in recent years. Based on the theories of systems biology, bioinformatics, proteomics, genomics and other disciplines, network pharmacology uses high-throughput omics data analysis, network database retrieval and computer simulation to reveal the network relationship of medicine-gene-target-disease interaction. By delineating these complex networks, network pharmacology can forecast the precise mechanisms of medicine action in disease treatment, assess medicine efficacy and potential adverse effects, and ultimately identify therapeutic strategies that are both effective and minimally toxic (Hopkins, 2008; Hong, et al., 2017a; Wang, et al., 2017; Zhang, et al., 2019; Nogales, et al., 2022). In addition, the integrated and systematic characteristics of network pharmacology coincide with the holistic concept of TCM and the idea of dialectical treatment, and it has been widely used in the screening of active compounds, the pathogenesis of diseases, and the interpretation of the specific mechanism of action of TCM in the treatment of diseases (Hao da and Xiao, 2014). Network pharmacology is based on the “disease-gene-target-medicine” interaction network to systematically observe the intervention and influence of medicines on the disease network, which is in the same direction as the principle of multi-component, multi-target and multi-pathway synergism of TCM compounds. The network pharmacology of TCM is the product of the development of the times and the inevitable trend of the modernization of TCM. The application of network pharmacology in the field of TCM can greatly solve the difficulties encountered in the modernization of TCM and open up a new way for the modernization of TCM. This research method is also fully in line with the holistic view and “treatment based on pattern differentiation” thinking of the Chinese medicine model, so network pharmacology has been widely used in the field of Chinese medicine research. And it is worth our attention that, precisely because of the deep integration of Chinese herbal medicine and network pharmacology, the concept of network ethnopharmacology (Fang, et al., 2005; Chandran and Patwardhan, 2017) is rapidly emerging to more clearly elucidate the effects of Chinese herbal bioactive substanceson both the interactome and the diseasome level of the organism. In this paper, we used “Traditional Chinese medicine”, “Non-alcoholic fatty liver disease” and “Network pharmacology” as the main keywords. By searching many databases such as Pubmed, Web of science and Embase, we finally screened and categorized the basic experimental studies related to the treatment of NAFLD with TCM in the last 10 years and with high quality of literature. For example, Pubmed’s algorithm is " ((((((Traditional Chinese medicine) OR (Chinese medicine)) OR (Herbal medicine)) OR (TCM))) AND ((((Non-alcoholic fatty liver disease) OR (NAFLD)) OR (Metabolic-associated fatty liver disease)) OR (MAFLD))) AND (Network Pharmacology)". We delved into the specific mechanism of action of TCM in the treatment of NAFLD, with a view to identifying problems in related research areas and providing new references for further studies in the future (Figure 2).

## Databases and research routes of network pharmacology

### Databases of network pharmacology

#### Disease databases

The DisGeNET (https://www.disgenet.org/) database (Piñero, et al., 2020) contains information about diseases and associated genes and mutation sites. With a collection of millions of gene-disease relationship pairs, containing 21,671 genes and 30,170 diseases, clinical or abnormal human phenotypes, DisGeNET has evolved over the past few years into different formats and tools that can be used with the database through a range of bioinformatics tools.

As one of the most commonly used comprehensive databases, GeneCards (https://www.genecards.org/) database (Stelzer, et al., 2016) automatically integrates approximately 150 web-based sources of gene-centric data, including transcriptome, proteome, genomic, genetic, clinical, and functional information, providing comprehensive, user-friendly information on all annotated and predicted human genes. The gene names include official names and aliases, and more importantly, GeneCards combines descriptions of genes from other online databases.

The OMIM (https://www.omim.org/) database (Hamosh, et al., 2005) is an authoritative database of human genes and genetic phenotypes, which is generally available for free retrieval. The data is comprehensive and timely updated, containing information on all known Mendelian diseases and more than 16,000 genes, focusing on the relationship between disease phenotypes and pathogenic genes, and providing information on clinical characteristics and pathogenesis of diseases. However the disadvantage is that the amount of gene data is relatively small, which needs to be further improved.

However, at the same time, we should note that when searching disease databases, different pathological stages of NAFLD, such as simple lipid accumulation, NAFL, and liver fibrosis, should be distinguished when appropriate, which can help to make the results of network pharmacology studies more precise.

#### Chinese medicine databases

TCMSP (Traditional Chinese Medicine Systems Pharmacology, https://old.tcmsp-e.com/tcmsp.php)(Ru, et al., 2014) is a pharmacological platform that integrates pharmacokinetics, pharmacochemistry, and the medicine-target-disease network. The database integrates information from PubChem (Kim, et al., 2019), PharmGKB(Barbarino, et al., 2018), and TTD (Wang, et al., 2020a) databases, including chemical constituents, targets, and medicine-target networks. As the most commonly used TCM database in network pharmacology, its biggest advantage is that it provides comprehensive evaluation data of human absorption, distribution, metabolism, and excretion (ADME) for each compound, involving pharmacokinetic properties of natural compounds such as drug similarity, oral bioavailability, blood-brain barrier, intestinal epithelial cell permeability, and water solubility. At the same time, the target of the potentially active molecule and its disease information are also provided. Therefore, users can select compounds with good drug similarity and ADME properties for further study.

TCMID (Traditional Chinese Medicine Integrated Database, https://www.bidd.group/TCMID/)database (Xue, et al., 2013; Huang, et al., 2018) includes six data areas, namely, disease, medicine, target, component, Herbal and compounding. The database information is collected by literature mining and citing other database information. This database connects TCM components with some disease databases (such as DrugBank (Wishart, et al., 2018), etc.) to establish the corresponding relationship between medicine components and diseases.

BATMAN-TCM (Bioinformatics Analysis Tool for Molecular Mechanism of Traditional Chinese Medicine, http://bionet.ncpsb.org/batmant-tcm/) database (Liu, et al., 2016) from TCMID, known medicines, compounds, and targets from DrugBank, Kyoto Encyclopedia of Genes and Genomes (KEGG) and TTD database, Users can search for the chemical constituents and corresponding targets of each TCM, and obtain the functional analysis results of these targets, including gene ontology (GO), KEGG pathway, OMIM, and TTD disease enrichment analysis results.

#### Functional databases

The STRING (https://string-db.org/) database (Snel, et al., 2000; Szklarczyk, et al., 2019) is mainly used to study protein interaction networks. The database is comprehensive, covering more than 5,000 species, more than 24 million proteins, and over 3 billion PPI relationships. STRING database can also be searched by protein sequence, protein name and other formats. For some individual proteins, STRING can also provide the network diagram composed of proteins that can interact with the protein, which can be used to mine the interaction between a single protein and other proteins; however, for multiple proteins, it can get the interaction network diagram among input proteins, which can be used to mine the interaction between multiple proteins.

DAVID (https://David.ncifcrf.gov/tools.jsp) database (Huang, et al., 2009) is also a comprehensive functional annotation analysis tool, first, upload the gene list, then use the functional annotation tool of the david database for further annotation of genes. This tool provides a rich analysis of genes in terms of biological pathways, protein-protein interactions, literature, and disease associations. DAVID functional annotation clustering tool can group redundant, similar, or heterogeneous annotation items into annotation groups based on the common association of genes in different annotation items, which is widely used in network pharmacological research.

The KEGG (https://www.genome.jp/kegg/) database (Kanehisa and Sato, 2020), a free and comprehensive database, enables further processing of biological processes, construction of appropriate modules and graphing, leading to a systematic analysis of the function of genes. The most important feature of KEGG is its powerful graphic function, which uses intuitive graphics to introduce the relevant signaling pathways and the intrinsic connection between each pathway, so that the researcher can have an intuitive and comprehensive understanding of the signaling pathways he/she wants to study.

GO (http://geneontology.org/) database (Ashburner, et al., 2000; Thomas, 2017), as one of the largest gene function information resource databases in the world, describes gene products from three aspects: cellular components, molecular functions and biological process. GO analysis is commonly used to find genes enriched in a particular cellular component, molecular function, and biological process. Nowadays, GO has a very broad application prospect, which can be used to integrate genetic information of different organisms, and can also be used to further predict disease-related genes and determine the function of protein structural domains.

#### Chemistry and compound databases

The ChEMBL (https://www.ebi.ac.uk/chembl/) database (Papadatos and Overington, 2014; Mendez, et al., 2019) is an online, free database that contains a wealth of ADME information, functions of bioactive compounds, etc., and the specific bioactivity data are derived from the corresponding literature, which is reliable and can be traced to the source of the data. We can search for specific information about a compound or target by name, molecular structure, sequence, etc., as well as information about the biological activity of a compound tested on the corresponding target.

The STITCH (http://stitch.embl.de/) database (Kuhn, et al., 2008; Szklarczyk, et al., 2016) contains information on predicted and known interrelationships of proteins with chemical components that include direct or indirect associations. This database has a large amount of data and also has a structural similarity comparison function that can identify chemical components with similar molecular structures by entering the chemical molecular structures of the components. Data sources for the STITCH database are interspecies knowledge conversion, text mining, computerized prediction, and integration of other databases. The main databases of network pharmacology involved are introduced as follows (Table 1).

**TABLE 1:** Public databases related to network pharmacology.

Type		Name	Description	Website for database or tool	References
Databases	Disease-related databases	SymMap	A database of 21,671 genes and 30,170 disease, clinical or abnormal human phenotypes with millions of gene-disease relationship pairs	https://www.disgenet.org/	Piñero et al. (2020)
		GeneCards	A web-based data source capable of automatically integrating about 150 gene-centered data sources, and also capable of predicting information about human genes	https://www.genecards.org/	Stelzer et al. (2016)
		OMIM	An authoritative database of human genes and genetic phenotypes, capable of providing information on the clinical characteristics and pathogenesis of diseases	https://www.omim.org/	Hamosh et al. (2005)
	Chinese medicine-related databases	TCMSP	A analysis platform and database that can retrieve information on key targets and components of Chinese medicine	https://old.tcmsp-e.com/tcmsp.php	Ru et al. (2014)
		TCMID	A Chinese medicine database that links Chinese medicine components to disease databases to establish the corresponding relationship between drug components and diseases	https://www.bidd.group/TCMID/	Huang et al., 2018; Xue et al., 2013
		BATMAN-TCM	A database capable of searching for the chemical constituents and corresponding targets of TCM and obtaining the results of the functional analysis of these targets	http://bionet.ncpsb.org/batmant-tcm/	Liu et al. (2016)
	Functional databases	STRING	A database primarily used to study protein interaction networks, it is also capable of being searched by protein sequence, protein name, and other formats	https://string-db.org/	Snel et al., 2000; Szklarczyk et al., 2019
		DAVID	A database with comprehensive functional annotation analysis that allows further annotation of genes after uploading a list of genes	https://David.ncifcrf.gov/tools.jsp	Huang et al. (2009)
		KEGG	A database that enables further processing of biological processes, construction of corresponding modules and graphs, and thus systematic analysis of the functions of genes	https://www.genome.jp/kegg/	Kanehisa and Sato (2020)
		GO	A database of gene function information resources capable of characterizing gene products in terms of cellular components, molecular functions and biological processes	http://geneontology.org/	Thomas, 2017; Ashburner et al., 2000
	Chemistry and compound databases	ChEMBL	An online free database containing a wealth of ADME information, functions of bioactive compoundsetc.	https://www.ebi.ac.uk/chembl/	Mendez et al., 2019; Papadatos and Overington, 2014
		STITCH	A database containing information on predicted and known interrelationships between proteins and chemical components with massive amounts of data	http://stitch.embl.de/	Kuhn et al., 2008; Szklarczyk et al., 2016

#### Research routes of network pharmacology

**FIGURE 3:**
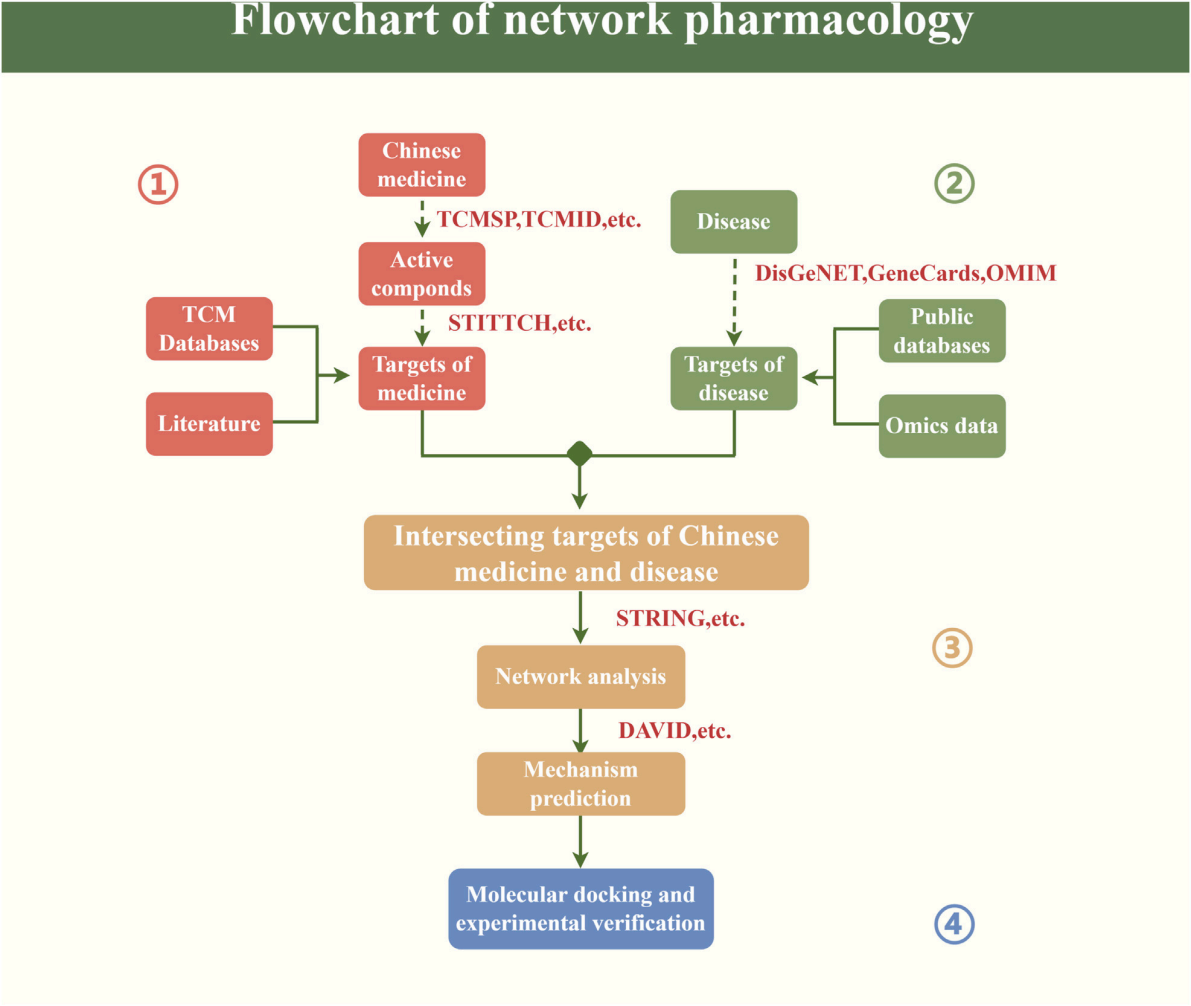
Flowchart of network pharmacology.

Network pharmacology is a burgeoning field that utilizes data analysis, computational virtual calculations, and network database retrieval to conduct extensive analyses of biological systems. This approach allows for the prediction of medicine componds and targets in order to enhance the efficacy of drug discovery. The research routes of network pharmacology is shown in Figure 3.

First of all, we should obtain a variety of key elements such as the composition and target of the Chinese medicine from relevant Chinese medicine databases or literature. As we all know, the composition of TCM is relatively complex, and further analyzing its metabolic process in the human body, clarifying the target of its final action as well as the composition of its entry into the bloodstream are the prerequisites for the study of the mechanism of action of TCM, and also indispensable factors in the process of future innovation and development of the database of TCM. Second, the corresponding targets of the disease are obtained from disease databases and related omics data. With the innovation and development of omics technology (Wu, et al., 2023a), genomic, proteomic, transcriptomic and other data will be more accurate and comprehensive to understand the gene regulatory mechanisms of diseases, providing new guidance and reference for the precise treatment of TCM. Again, based on the above analysis, the interrelationships among the elements are speculated, the key targets of medicines and diseases are intersected, and further network analysis and mechanism prediction are carried out, so as to study the key mechanisms of action of TCM in treating diseases, as well as the pharmacological characteristics and so on (Li, et al., 2023a). Finally, suitable key components and core targets were selected for molecular docking, so as to further explore the binding sites and pockets of key components and core targets (Stanzione, et al., 2021). Meanwhile, the acquired potential active compounds and targets can also be verified by relevant animal experiments or cellular experiments at a later stage.

## Network pharmacological study on non-alcoholic fatty liver disease treated by traditional Chinese medicine

### Single Chinese medicine and its extract

#### Heat-clearing medicines

Chinese herbal medicine *Andropanthes* is a typical heat-clearing medicine, with heat-clearing detoxification, cooling blood, detumescence, and dry dampness effect, its medicine is bitter cold, should not be taken for a long time, take more. A study (Dai, et al., 2019) found that andrographolide, as one of its main active compounds, has very rich pharmacological activities, mainly including anti-diabetes, anti-inflammatory, anti-obesity, anti-cancer, and so on. Through network pharmacology and molecular docking technology, Li and his research team (Li, et al., 2021) found that andrographolide plays a role in the treatment of NASH mainly through antioxidant, anti-inflammatory, and regulation of new fatty acid synthesis. The core targets enriched in the signaling pathway of NAFLD mainly include IL1B, MAPK8, AKT1, IL6, and TNF, etc., which further provides an important scientific basis for demonstrating the mechanism of andrographololides against NASH. At the same time, the results of such research also revealed the key mechanism of “multi-target, multi-pathway” in the treatment of chronic diseases by TCM.

Black ginseng (BG), the product of TCM, is ginseng with clearing fever and detoxification, deswelling and diuresis, as well as anti-inflammation and antibacterial. BG is mostly made of fresh ginseng as raw materials, tea brown or dark brown ginseng products made by repeated steaming and drying, and its eating methods generally include boiling soup, grinding powder, etc. Some relevant studies (Hu, et al., 2017; Choudhry, et al., 2019) have found that BG can effectively prevent liver injury, but there are relatively few studies on the pharmacological mechanism of BG in the treatment of NAFLD, which is worth further exploration in the future. Some studies (Wei, et al., 2022) have found that the core target of BG in the treatment of NAFLD is AKT1, and it has a good binding affinity with ginsenosides, the main component of BG, and experimental verification has found that BG administration can improve the liver histological changes induced by methionine and choline deficiency diet. The levels of serum aspartate aminotransferase (AST) and alanine aminotransferase (ALT) can be effectively reduced. At the same time, BG can also upregulate the phosphorylation level of AKT in the liver of NAFLD mice, thus further exerting its therapeutic effect on NAFLD.

Pure total flavonoids from Citrus (PTFC) are mainly derived from grapefruit peel, and Narirutin, neohesperidin and naringin are the three main components of PTFC. It has a significant effect on the prevention and treatment of liver disease (Wu, et al., 2017). Hupomelo peel is very rich in nutritional value, its cool, with a certain effect of clearing heat and detoxification. Moderate consumption of Hupomelo peel can effectively remove the heat toxin accumulated in the body and relieve the phenomenon of constipation or oral and tongue sores. And Hupomelo peel also has a certain effect of relieving cough and phlegm, and has an auxiliary therapeutic effect on sore throat and cough and phlegm. In addition, the amino acid content of Hupomelo peel is very high, in addition to containing trace elements and vitamin c, can enhance capillary permeability, supplement the human body needs trace elements. Hong et al. (Hong, et al., 2019) found that the vascular endothelial growth factual-C (VEGF-C) was a key target of PTFC in the treatment of NAFLD through network pharmacological analysis. Further studies have shown that VEGF-C is also involved in cytokine-cytokine receptor interaction and Focal adhesion pathway in the PTFC treatment network for NAFLD, and COL4A1 is related to focal adhesion pathway. It is concluded that VEGF-C and COL4A1 play important regulatory roles in the treatment of NAFLD.

*Lonicera japonica Flos* (LJF), as one of the representative medicines of clearing heat and detoxifying Chinese medicine, is a dry bud or first blooming flower of *Honeysuckle* with cold nature, which has the effect of clearing heat and detoxifying and dispersing wind and heat. Pharmacological studies have confirmed that LJF has ideal therapeutic effects on infectious diseases and inflammation, and has outstanding effects on multiple targets *in vivo* and *in vitro* (Li, et al., 2020). By mapping the target of the key active compound in LJF with the target phase involved in NAFLD, some scholars (Sun, et al., 2022b) predicted the main key target of LJF’s participation in its anti-NAFLD activity, and finally predicted 17 active compounds in LJF and 29 main targets related to NAFLD, and the molecular interconnection results showed that, Isochlorogenic acid B can stably bind to CASP3 and TNF-α. Finally, through experimental verification, it was found that LJF can significantly downregulate the expression of CASP3 and TNF-α in the NAFLD cell model, and finally play a therapeutic role in NAFLD. The above herbal remedies for clearing heat have a significant effect on the treatment of NAFLD, which provides an important preliminary basis for further exploration in the future.

#### Deficiency tonic medicine

Chinese herbal medicine *Paeoniae Radix Alba* (PRA) is a tonic medicine for deficiency, originally published in Shennong Herbal Classics. It is the dried root of the Peony in the ranunculus family. It has the effects of nourishing blood and regulating the menstrual period, collecting Yin and stopping perspiration, soothing the liver and relieving pain, and calming liver-yang. Relevant scholars (Shu, et al., 2014) have found that PRA has significant properties of tonic, astringent, analgesic, and anti-spasticity, and is widely used in clinical practice. In recent years, PRA has played an increasingly important role in the treatment and prevention of liver-related diseases such as acute liver injury and NAFLD, which deserves our in-depth and extensive research (Qin and Tian, 2011). Luo et al. (Luo, et al., 2021) proposed a comprehensive strategy for rapid identification of active compounds in food and medicinal plants based on network pharmacology, molecular docking, sequential metabolite identification methods, and surface plasmon resonance (SPR) analysis, and finally screened 377 related targets and 31 bioactive candidates. It was found that the metabolic pathways related to PRA alleviating NAFLD were mainly methylation, gluconaldehyde acidification, sulfation, and oxidation, providing a new perspective for the study of PRA treatment and prevention of NAFLD.

*Eucommia ulmoides* has the effect of tonifying the liver and kidney, strengthening muscles and bones, and calming pregnancy. It is widely used in clinical practice. It can also be used in wine, water, or soup. Related *in vitro* and *in vivo* studies have found that *eucommia ulmoides* monomer compounds and extracts have a wide range of pharmacological effects, especially in hyperlipidemia, obesity, aging, sexual dysfunction, diabetes, hypertension, and immune regulation (He, et al., 2014). *Eucommia ulmoides* leaves have been included in the Chinese Pharmacopoeia. Gong et al. (Gong, et al., 2022) used network pharmacological methods to predict the potential effective components and specific mechanism of action of *Eucommia ulmoides* leaves in regulating NAFLD, and further conducted *in vitro* experimental verification to explore its lipid-lowering effect and mechanism. Finally, it was found that its main components and core proteins regulating lipid metabolism, especially flavonoids, had a good binding degree, and EUL 50 could significantly reduce lipid accumulation in the body, increase the expression level of PPARγ, and finally exert its therapeutic effect on NAFLD. Moreover, it was found that the main effective components regulating NAFLD were phenols and flavonoids. It provides a solid foundation for future research.

*Gynostemma pentaphyllum* is a grassy climbing plant belonging to the family of gourd. With a bitter taste and cold flavor, *gynostemma pentaphyllum* has the functions of clearing heat and detoxifying, relieving cough and expelling phlegm, nourishing the heart and soothing the mind, etc. It is a common medication for patients with faltering symptoms (Niu, et al., 2013). In cell experiments and animal experiments *in vitro* and *in vivo*, Gynostemma pentaphyllum is proven to have a variety of biological activities, including liver protection, anti-cancer, anti-dementia, anti-atherosclerosis, and lipid regulation (Su, et al., 2021). Through PPI analysis, GO analysis, and KEGG signaling pathway enrichment analysis, Wang et al. (Wang, et al., 2022) screened out key therapeutic targets and pathways and specific mechanisms of action, and found that AKT1 and GSK3B were key therapeutic targets. The results of the enrichment analysis showed that the main mechanism is the biological process related to insulin resistance, and the P13K-AKT signaling pathway is the key pathway of this mechanism. This finding provides a new perspective for *Gynostemma pentaphyllum* to alleviate NAFLD in the future.

*Ganoderma lucidum* is a supplement with the same origin as medicine and food, containing rich nutrients, which can enhance the body’s immune ability, assist in the treatment of diseases, and have the effects of invigorating qi and calming, relieving cough and relieving asthma. Studies (Lin and Zhang, 2004) have confirmed that *G. lucidum* has the effect of regulating many components of the immune system, such as T lymphocytes, B lymphocytes, and NK cells. One of the major triterpenoids of *G. lucidum*, Resinacein S, has been shown to regulate lipid metabolism and mitochondrial biogenesis. Mao et al. (Mao, et al., 2023) found that Resinacein S can significantly improve lipid metabolism in liver cells, have a protective effect on steatosis, and can alleviate liver damage. PPI network analysis also found that its central protein can be used to characterize the main target of Resinacein S against NAFLD.

The TCM Schisandra chinensis originates from China, which can strengthen immunity and has high medicinal value. It has the effects of astringent and astringent, invigorating qi and promoting fluid, tonifying kidney, and calming the heart. *Schisandra chinensis* fruit has been widely used in the treatment of chemically active viral liver injury with remarkable efficacy (Rybnikář, et al., 2019; Yang, et al., 2022a). Li (Li, et al., 2023b) uses network pharmacology to predict key compounds, potential therapeutic targets, and related signaling pathways and finds through *in vitro* experiments that the key components of *S. chinensis* fruit ethanol (EtOH) extract are mainly concentrated in the soluble fraction of petroleum ether (PET). Network pharmacological analysis found that the mechanism of NAFLD remission mainly lies in the main active lignans contained in PET, and these lignans can regulate insulin resistance signaling pathways, thus playing a therapeutic role. Finally, through *in vivo* experiments, it was found that PET and core active compound 3 treatment significantly alleviated liver steatosis and reduced the levels of AST, ALT, TG and total cholesterol (TC) in mice, and finally confirmed its significant regulatory effect on NAFLD.

#### Blood-activating medicine

As one of the traditional medicines for promoting blood circulation and removing blood stasis, saffron has been favored by the majority of doctors since ancient times. It has the effect of promoting blood circulation and removing blood stasis, relieving depression and calming nerves, protecting the liver and gallbladder, and has high medicinal value. It is a very precious medicinal material. Foreign scholars (El Midaoui, et al., 2022) have found that saffron is rich in terpenes and carotenoids, and has strong anti-inflammatory, anti-tumor, and antioxidant effects *in vivo* and *in vitro*. Through network pharmacology and experimental verification, Xu et al. (Xu, et al., 2021) found that saffron contains a total of 206 key components, including crocetin (CCT), and also contains 6 cross-targets, mainly AKT1, MAPK1, and STAT3, etc., while the AGE-RAGE signaling pathway in diabetic complications, HIF-1, TNF, and other diabetes-related signaling pathways are the main signaling pathways involved in the therapeutic effects of saffron on NAFLD. Further studies found that CCT significantly reduced the activities of ALT and AST, as well as the levels of TG, creatinine, malondialdehyde, TC, blood urea nitrogen, and uric acid, which prevented further exacerbation of NAFLD, and its main mechanism of action was closely related to the upregulation of HO-1 and Nrf2 expression, the reduction of inflammation, and the inhibition of oxidative stress.

*Radix salviae miltiorrhizae* (Danshen in Chinese) is a perennial upright herb of the cheilaceae family and Salshen genus. It likes the environment of mild climate, sufficient light, moist air, and fertile soil. It is one of the main representatives of medicines for promoting blood circulation and removing blood stasis. The effect of clearing the heart and eliminating irritability, cooling blood, and eliminating carbuncle. Nowadays, the emerging network pharmacology, omics analysis, chemical analysis, and molecular pharmacognosy make the modern research of Danshen and rhizomes further in-depth. Studies (XD, et al., 2019) have reported that Danshen has more than 200 related compounds, including water-soluble phenolic acids, lipophilic diterpenoids, and other major components, and has shown a variety of pharmacological activities such as anti-tumor, anti-oxidation, anti-atherosclerosis, anti-inflammation, anti-diabetes. *In vivo*, Danshen was found to be effective in reducing steatosis and liver inflammation in animal models of NAFLD. Furthermore, network pharmacology was further applied to find that six potential key components, including Danshensu, salvianolic acid b, and tanshinone iia, may play a role in protecting the liver and regulating intracellular molecular targets such as PPARα, MMP2, and CYP1A2. These potential key components regulate the body’s lipid metabolism, anti-fibrosis, and anti-oxidation, and ultimately exert the effect of NAFLD treatment (Hong, et al., 2017b).

Blood activating medicine *Paederia scandens* has attracted much attention since ancient times. It has the effect of dispelling wind and dampness, relieving pain and detoxification, dissipating food and removing accumulation, promoting blood circulation, and eliminating swelling. *Paederia scandens* has been widely used in Vietnam, Japan, and India, and some studies have found that its extract has many biological activities such as anti-cancer and anti-virus (Trung, et al., 2023). Wu et al. (2021) successfully constructed a “medicine-compound-target-disease” network by using network pharmacology methods, and further experimental verification confirmed that *P. scandens* could significantly downregulate serum TG levels in NAFLD chickens, and through toll-like receptor signaling pathway, The *Salmonella* infection pathway and apoptosis pathway regulate the liver cell cycle and apoptosis and finally exert their therapeutic effects.

*Polygoni Orientalis Fructus* (POF) is a well-known medicinal and ornamental plant with a good blood-activating effect, good antioxidant activity (Lei, et al., 2005), and certain anticancer potential (Shan, et al., 2022). Some scholars (Chen, et al., 2021) conducted an in-depth exploration of the specific mechanism of action of POF, conducted network pharmacological analysis, and screened out a total of 18 key components in POF, including 277 potential targets. Moreover, statistical pattern recognition was further used to analyze the quantitative data matrix of medicine, disease and target, and NAFLD was screened out from a variety of candidate diseases. Eventually, it became the best indication for POF. At the same time, *in vitro* studies have also found that POF can effectively activate the AMPK signaling pathway, inhibit the NF-κB signaling pathway, relieve free fatty acid-induced inflammation, oxidative stress, steatosis, mitochondrial dysfunction, etc., and finally confirm that POF can be used as a special key therapy for NAFLD.

The Chinese herbal medicine *artichoke* is cool and has the unique effect of promoting blood circulation, diuresis, etc., it is rich in nutrition, high edible value, and the purpose of curing alcohol and protecting the liver, but during the consumption of artichokes, you should pay attention to a reasonable diet, do not ingest cold, spicy and irritating food, so as not to affect its efficacy. As one of the key components of artichoke, *Cynarine* has been valued by many scholars. Sun and their research team (Sun, et al., 2022a) found that there were 48 intersection targets of *Cynarine* and NAFLD, among which TP53, ELANE, MMP9, CASP3, and NOTCH1 were the core therapeutic targets. Through the PPI network, inflammation and immune-related targets played a key therapeutic role. In addition, it was found that *Cynarine* was mainly designed to treat NAFLD with the PI3K-Akt signaling pathway and MAPK signaling pathway, and it was finally concluded that Cynarine could significantly reduce the fat deposition capacity of NAFLD model cells. The AST and ALT levels released by hepatocytes due to excessive lipid accumulation are downregulated.

#### Liver clearing and eye clearing medicine

*Cassiae semen* (CS) has clear liver, bright eyes, laxative effect, the elderly drinking cassia tea not only helps smooth stool, but also can regulate fat, blood pressure, generally more decoction, can also be used for external use, but also tea, porridge, the effect is very obvious. Studies (Dong, et al., 2017) have found that the main extracts of CS have anti-hyperlipidemia, liver protection, neuroprotection, antibacterial, anti-diabetic, antihypertensive and antioxidant effects, and have been widely used in clinical practice. Network pharmacology studies (Huang, et al., 2023) have found that CS treatment of NAFLD mainly involves the key targets of PIK3CA, CASP3, A4, EGFR, APP, and reduces the expression of CASP3 and EGFR by regulating MAPK signaling pathway, significantly reducing intracellular lipid accumulation, and finally exerting the effect of regulating NAFLD. It lays a foundation for the future study of CS.

As one of the most common chronic liver diseases, NAFLD is mainly characterized by excessive accumulation of fat in the liver. As one of the commonly used foods or medicines for liver diseases, blueberry has a good effect on clearing the liver and improving eyesight. However, its specific mechanism of action in the treatment of liver diseases is worth further exploration. Wang, et al. (2021a) established the TCM-compound-target network through Cytoscape, made the protein-protein interaction network (PPI), obtained 53 key targets and 4 important compounds, and again confirmed by cell experiments. blueberry leaves (PBL) can rapidly improve the viability of NAFLD cell models and further found that the protein expressions of Bcl-2 and Caspase-3 were in line with the expected mechanism of action of PBL, finally revealing that the multi-target mechanism of PBL against NAFLD is closely related to apoptosis pathways.

As one of the most commonly used Chinese medicines for the treatment of NAFLD, *Silybum marianum* is often used in a variety of liver-protecting proprietary Chinese medicines. It has the effect of soothing the liver and improving the gallbladder and is widely used. Scholars such as Abenavoli, et al. (2018) have found that *S. marianum*, as the main component of silybin, also has anti-fibrosis, liver regeneration, antioxidant, immunomodulatory and anti-inflammatory properties. Jiang, et al. (2022) used TCMSP, DisGeNET, UniProt database, and Venny 2.1 software to identify 11 key active compounds, 92 gene targets, and 30 core liver protection gene targets, respectively. It was confirmed that *S. marianum* decreased AST, ALT, TG, TC, and low-density lipoprotein cholesterol levels, downregulated the protein expression of MAPK1, IL-6, p53, Caspase 3, and VEGFA, and increased the protein expression of AKT1. Ultimately, it plays a role in protecting the liver and preventing the development of NAFLD.

#### Herb pair of medicine

Herb pair refers to the combination of two flavors of herbs together, mostly synergistic or attenuated effects, most of the two flavors of medicines together take the form of medication, and are the smallest fixed unit in the compatibility of TCM. The medicine pair has the characteristics of simple medicine use, profound theory, and specific medicine force, and its purpose is to obtain a better therapeutic effect than using a single herb. Huanglian-Wuzhuyu herb pair (HWHP) is a classical Chinese medicine pairing, which has been often used in clinical treatment since ancient times, such as the zuojin pill, with remarkable efficacy. Zhang, et al. (2022) studied the specific mechanism of action of HWHP in the treatment of liver disease through network pharmacology and conducted *in vivo* experimental verification. A total of 41 key compounds and 51 targets related to NASH were screened out, suggesting that HWHP may alleviate NASH by controlling the inflammatory response. Through experimental verification, it is finally concluded that HWHP can reduce the occurrence of liver steatosis and inflammation by inhibiting the NLRP3 inflammatome, thereby improving the NASH of mice and playing a role in preventing liver diseases.

#### Traditional Chinese medicine compound

Patients with NAFLD may have different symptoms, so it is necessary to combine the specific syndrome type to conduct symptomatic treatment. Therefore, different traditional prescriptions for NAFLD with different syndrome types are effective. Nie et al. (2020) conducted GO enrichment analysis and protein interaction (PPI) analysis of Chaihu Shugan powder (CSP) based on network pharmacological methods of multiple databases. Molecular docking technology was used to screen nuclear receptors and further determine the key components of CSP. FXR, PPARα, PPARγ, and RARα were finally selected as the key targets, and naringin, isorhamnetin, kaempferol, and quercetin were selected as the key compounds. In the experimental validation, after CSP treatment, the liver histopathology, liver lipids, body weight, and serum of NAFLD rat models were significantly improved, and the mRNA levels of FXR and PPARγ were significantly changed, reflecting the good intervention effect of CSP on NAFLD rats. Xie and his research group (Xie, et al., 2022) also found that CSP inhibits angiogenesis and modulates the inflammatory response in the liver, and also has a significant anti-fibrotic effect that can effectively prevent the progression of NAFLD to liver cirrhosis. Wei, et al. (2021) conducted an in-depth study on the traditional prescription Sinisan (SNS) and used the network proximity algorithm to evaluate the main efficacy of SNS on NAFLD. The final results showed that the average shortest path length between SNS and NAFLD genes was significantly smaller than the degree-matched random ones. Through experimental verification, SNS can reduce liver steatosis and liver inflammation, and inhibit the occurrence of hyperlipidemia. Wang, et al. (2021b) found through network pharmacological analysis that SNS can effectively alleviate liver fibrosis by reducing inflammation, hepatocyte apoptosis, ECM accumulation, and abnormal angiogenesis, and hinder the further development of liver diseases. A recent *in vivo* and *in vitro* experimental study (Lan, et al., 2024) found that SNS was able to attenuate lipid accumulation in hepatocytes and mouse liver by modulating the AMPK/p300/SREBP-1c axis, thereby inhibiting the expression of fatty acid synthase. And Jiang, et al. (2023) found that SNS can significantly improve the progression of fibrosis and prevent hepatocyte apoptosis in mice with liver fibrosis, and its anti-apoptosis mechanism is closely related to the key component isorhamnin’s inhibition of AKt-mediated downregulation of FXR expression. Shengmai-Yin and Ganmaidazao decoction (SGD) were both TCM prescriptions, which were widely used clinically. Li et al. (2019) explored it deeply by using the network pharmacology method and conducted relevant experimental verification. It was finally confirmed that SGD can effectively reduce the number of coronary structures in visceral adipose tissue, reduce the size of adipocytes, and reduce hepatic lipid deposition. At the same time, SGD can also improve liver metabolism by increasing the expression of HSL, PI3K/Akt, and PPARα, and decreasing the expression of FASN and SREBP-1. Shenqi pill (SQP) is one of the most commonly used classical prescriptions for tonifying the kidney and has long been used to treat kidney-yang deficiency syndrome. By using bioinformatics and network pharmacology, He, et al. (2022) found that CDKN1A, JUN, MYC, and PTGS2 may be the key targets for SQP to improve inflammatory response and treat liver diseases. Moreover, experimental validation results showed that lipid metabolism and liver damage indexes of NASH rats treated with SQP were significantly improved. It is suggested that it has a good therapeutic effect on liver disease.

Traditional prescription Erchen decoction (ECD) is a phlegm-removing prescription, which has the effect of dryness, phlegm removal, and qi regulation. Liu et al. (2021) further determined the key compounds of ECD and its potential therapeutic target for NAFLD by using bioinformatics and network pharmacology methods. Finally, it was confirmed that ECD reversed the levels of inflammatory factors such as TNF-α, serum LPS, IL-1β, and liver TLR-4, which could further alleviate liver inflammation and achieve the effect of treating NAFLD. A recent ethnopharmacology study (Deng, et al., 2024) used lentivirus-mediated transfection techniques to establish Caveolin-1 knockdown cell lines and found that ECD was effective in promoting Caveolin-1 expression, ultimately alleviating iron and lipid accumulation in NAFLD mice.

Erzhi pill (EZP), as a classic Chinese medicine prescription, has a potential hepatoprotective effect on NAFLD. Huang, et al. (2020) established a systematic analysis platform to explore the specific mechanism of EZP on NAFLD and the key compounds involved. Finally, it was found that EZP exerts an anti-NAFLD effect through multi-target, multi-component, and multi-pathway, and linarin and luteolin may be the key compounds of EZP in the treatment of NAFLD. Fuzi Lizhong decoction (FLD) originates from ancient Chinese pharmacopoeia and has been applied clinically for many years. Yang, et al. (2020) explored deeply the key potential mechanism of FLD treating NAFLD by using the method of network pharmacology. It is found that FLD may act on NAFLD by regulating PPARG and p53, which has been further confirmed *in vivo* and *in vitro*. At the same time, FLD can also effectively reduce the increase of serum TG, TC, liver-free fatty acids, and blood sugar levels caused by NAFLD. Ganlu powder (GLP) is also a classic Chinese medicine formula, which has good effects on the treatment and prevention of chronic liver disease. Gao, et al. (2022) found through the network pharmacological study that GLP may treat nonalcoholic steatohepatitis by regulating phosphatidylinositol 3-kinase signaling and inflammatory response, and ultimately achieve therapeutic effect by influencing the TNF signaling pathway through AKT1. The famous traditional decoction Xiaochaihu decoction has been proven to have the effect of treating NAFLD, but its mechanism remains unclear. Therefore, Hu, et al. (2020) explored the specific mechanism of Xiaochaihu decoction treating NAFLD by using the network pharmacological method. Among them, oxidative stress regulation, metabolic regulation, and immune regulation are the key regulatory cores in the mechanism of action. The results of meta-analysis (Zhu, et al., 2021) likewise confirmed that for the prevention and treatment of NAFLD, the safety and efficacy of Xiaochaihu decoction has been recognized by a wide range of scholars.

The classic prescription San-Huang Tang (SHT) has been clinically used in the treatment of diseases such as type 2 diabetes and obesity, and SHT has recently been proven to have a good therapeutic effect on NAFLD. Shi, et al. (2022) found through bioinformatic screening and *in vivo* and *in vitro* experimental verification that SHT further affects insulin resistance by activating INSR/IRS1/AKT/FoxO1 pathway, and finally exerts the therapeutic effect of NAFLD. This study not only provides a solid experimental basis for the therapeutic effect of SHT but also provides a solid experimental basis for the therapeutic effect of SHT. It also provides a new target for the treatment of NAFLD. ZeXie Decoction (ZXD) is a traditional decoction composed mainly of decoction, which can treat a variety of diseases clinically. Wu et al. (2021) used network pharmacology to study the association between NAFLD and ZXD established a complex network model of herbal-compound target-pathway and found that ZXD can exert a good therapeutic effect on NAFLD by regulating NF-κBp65, HMGCR, MAPK1, and SREBP-2. Chaihu Lizhong Tang (CHLZT) is derived from ancient Chinese medical classical works. Zhang, et al. (2020a) constructed the components-target network of “CHLZT” through Cytoscape software, and screened the core genes of CHLZT in the treatment of NAFLD through network topological parameters. CHLZT is mainly involved in the IL-17 signaling pathway, AGE-RAGE signaling pathway in diabetes complications, protease binding, and cofactor binding, and finally plays a potential regulatory mechanism for NAFLD.

#### Clinical experience prescription

Nowadays, in addition to traditional classical prescriptions, there are more and more clinical experience prescriptions for the treatment of NAFLD, which have very good effects and are well received by the majority of patients. It was found that Yiqi-Bushen-Tiaozhi (YBT) effectively alleviated diet-induced non-alcoholic steatohepatitis in C57BL/6 mice. Therefore, Hong, et al. (2020) applied Ingenuity Pathway Analysis (IPA) to combine miRNA and mRNA deep sequencing data for network pharmacology research. This study found that YBT may ultimately exert a therapeutic effect by regulating the expression of these miRNAs, which may regulate immune/inflammation and oxidative stress. YBT also rapidly ameliorates metabolic disorders in mice and exerts positive therapeutic effects on NASH mice by improving the abundance and diversity of intestinal flora (Yan, et al., 2022). In recent years, Jian Pi Qing Gan Yin (JPQGY) has been used in the clinical treatment of NAFLD in China, and some scholars (Liu, et al., 2022) have conducted network pharmacological analysis and experimental verification of it. JPQGY can significantly alleviate the steatosis-inflammation-fibrosis of NAFLD mice, effectively prevent the downregulation of Pparα and AMPK, and upregulate Nf-κb, F4/80, SREGBP-1C, Cyp2e1 and LXRα. The final study results provide sufficient evidence to support the clinical use of JPQGY in high-fat diet-induced NAFLD. Jiangzhi (JZD) Decoction is also an empirical prescription commonly used in the clinical treatment of NAFLD. Wang et al. (2020) found 147 active compounds, 1,285 core targets, 401 key NAFLD genes, and 59 overlapping targets of JZD treating NAFLD. Through PPI analysis, 22 core targets were obtained. The results of the study confirmed that JZD alleviates hepatocyte steatosis by regulating key molecules related to lipid metabolism and nuclear receptor transcription, such as HNF4α, LXRα, and PPARα, and finally exerts therapeutic effects. A recent metabolomic analysis (Wang, et al., 2023) also found that JZD was able to prevent the progression of NAFLD by modulating lipid metabolic pathways and restoring the levels of differential metabolites in rats in the high-fat diet group. The effect of Qushi Huayu Decoction (QHD) on treating NAFLD was also very significant. Gao, et al. (2020) used HitPick systems, the SEA, and the Swiss Target Prediction to search for the target of the key active compound. Finally, 128 key active components and 275 corresponding targets in QHD were obtained through screening, and 55 core targets and 27 important signaling pathways were further screened, such as the P13K-AKT signaling pathway, cancer pathway, PPAR signaling pathway, etc. The specific pharmacological mechanism of QHD in NAFLD was revealed. A recent lipidomics study (Ni, et al., 2023) also found that QHD modulates the gut microbiota, which in turn interferes with serum lipids and exerts an anti-NAFLD effect.

Qutan Huoxue decoction (QTHX) was clinically used to treat non-alcoholic steatohepatitis and had a good effect. Wu, et al. (2023b) found through network pharmacological study that The liver protective effect of QTHX is closely related to oxidative stress, anti-inflammatory response, and lipid receptor signaling, and QTHX has been shown to treat liver inflammation in rats by activating the SCOS1/NF-κB/TLR4 pathway. Xu, et al. (2022) conducted a comprehensive analysis of Shen-Shi-Jiang-Zhuo formula (SSJZF) by RNA sequencing and network pharmacology, and found 23 main compounds of SSJZF and 25 key therapeutic targets for NAFLD. SSJZF was proven to improve NAFLD in rats by activating the PI3K/Akt pathway. Another scholar (Yang, et al., 2022b) explored the potential bioactive compounds and specific mechanisms of Shugan Xiaozhi (SGXZ) decoction treating NAFLD through network pharmacology, molecular docking, and molecular dynamics simulation. It was found that the key active compound isochlorogenic acid A may directly bind to VEGFA, IL-6, RELA, and MMP9, and exert a therapeutic effect by regulating the PI3K-Akt signaling pathway. Through analyzing Shuangyu Tiaozhi Decoction (SYTZD), Yin, et al. (2022) also found that HIF-1α, FASN, GSK-3β, ESR1, mTOR, and VEGFA were the main targets for SYTZD to treat NAFLD. The main signaling pathways involved include the HIF-1 pathway, AMPK pathway, insulin resistance pathway thyroid hormone pathway, etc. It has been found through experiments that SYTZD can improve lipid deposition, insulin resistance, and inflammation to play a role in the treatment of NAFLD.

#### Chinese patent medicine

Fufang Zhenzhu Tiaozhi formula (FTZ) is a commonly used proprietary Chinese medicine for the treatment of liver diseases. Wang, et al. (2021c) found that the treatment of FTZ had a positive effect on liver fat accumulation and liver steatosis in the hiathological features of oil red O and HE staining and electron microscope examination. FTZ can also improve liver metabolism by decreasing HIF-1α expression and increasing phosphorylation of PI3K-AKT and ultimately play a regulatory role in NAFLD. FTZ was able to improve liver function and reduce TG levels in NASH mice, and it was also found that FTZ also improved intestinal barrier function, attenuated intestinal inflammatory response, and more effectively prevented hepatic inflammatory cell infiltration and liver fibrosis (Lan, et al., 2022). GanShuang Granules (GSG), derived from the classical Chinese formula Xiaoyao San, are mainly used in the clinical treatment of chronic liver diseases. Zhi, et al. (2023), by integrating the results of network pharmacological analysis and relevant experimental verification, found that GSG has a good therapeutic and preventive effect on NAFLD rats, mainly reflected in the inhibition of NF-κB/IκB signaling pathway and its downstream apoptotic and inflammatory signals. A recent study (Guoguo, et al., 2024) also found that GSG does have a good therapeutic effect on NAFLD mice, with a significant reduction in serum levels of inflammatory factors after GSG intervention, and the therapeutic targets involved are mainly closely related to the PI3K/AKT signaling pathway. Jian-Gan-Bao (JGB) is a functional herbal formula that is also commonly used in the treatment of liver diseases such as NAFLD. Some scholars (Li, et al., 2018) have identified 40 key compounds and 15 related targets of JGB through network pharmacological studies and confirmed that the specific mechanism of JGB in the treatment of NAFLD is closely related to inflammation, fatty acid oxidation, cell proliferation-related pathways, and tumor necrosis factor (TNF) production. In addition, relevant scholars (Zhou, et al., 2021) used Cytoscape software to build Jiangzhi Granule component-target-disease network, including 8 key components such as quercetin, resveratrol, and emodin, and 10 major targets such as IL2, TNF, and CCL2. It was proved that Jiangzhi Granule could reduce nonalcoholic steatohepatitis by inhibiting the M1-type polarization of macrophages mediated by the TNF/NFκB signal.

Chinese medicine compound Kangtaizhi granule (KTZG) granule has clinically proven to have an obvious therapeutic effect on NAFLD. Scholars such as Zhang, et al. (2020b) identified the major chemicals of KTZG and related targeted pathways for NAFLD therapy by using high-performance liquid chromatography and network pharmacological analysis methods, and found through experimental verification that KTZG can improve hepatic steatosis and lipid accumulation in rats and HepG2 cells fed by high-fat diet, and play a role in preventing the occurrence and development of NAFLD. Mai luoning Oral Liquid (MLN) is a modern Chinese medicine prescription composed of Dendrobium, Achyranthes, honeysuckle, and scrophulariae. Some scholars (Jia, et al., 2022) have found through network pharmacological analysis that the PPARα signaling pathway plays a key role in the progression of chronic liver disease in MLN. It was proved that MLN could significantly improve non-alcoholic steatohepatitis in mice. Hung, et al. (2021) confirmed the specific mechanism of action of PingTang No.5 capsule (PT5) in the treatment of NAFLD, conducted network pharmacological studies on it, and conducted experimental verification. They found that PT5 could significantly reduce the weight, fat cell size, and blood fatty acids of mice when administered to NAFLD mice. Finally, the metabolic function of mice was improved.

Some scholars (Tan, et al., 2023) applied serum pharmacochemical methods to identify the key components of Yinzhihuang granule (YZHG) granule, and predicted the core targets of YZHG granule in the treatment of NAFLD by systems biological methods. The results showed that YZHG granule might play a therapeutic role in enhancing intestinal barrier and improving intestinal flora destruction. Therefore, it can regulate liver lipid metabolism and reduce liver inflammation. Zeng, et al. (2020) also confirmed that Yinzhihuang liquid (YZHL) can effectively accelerate lipid β oxidation, relieve liver necrotic inflammation, inhibit lipogenesis and relieve oxidative stress, and finally achieve the effect of treating nonalcoholic steatohepatitis in rats. YZHL is also able to regulate the gut-liver axis to prevent the occurrence of NASH in mice, and play a role in reducing body weight, preventing obesity and alleviating hepatocyte steatosis (Li, et al., 2022). The above proprietary Chinese medicines have different degrees of efficacy in the treatment of NAFLD, and are widely used in clinical practice (Supplementary Table S1). In the future, we still need to carry out further research and exploration, and contribute to the development of clinical new medicines.

## Conclusions and perspective

In recent years, network pharmacology, as a new subject, has been widely used in the research of various fields of TCM. The development of network pharmacology and application to study the mechanism of action of TCM to treat disease provides a new way of thinking, different from the past “single medicine - single compound - single disease” of the linear model, but will be associated with the disease of medicine, composition, targets, and disease factors include all and comprehensive analysis, clearly reflects the composition and characteristics of targets and pathways, This treatment mode is consistent with the overall concept of TCM and is more consistent with the thinking mode of treating diseases in TCM. Based on the research methodology of network pharmacology, TCM achieves a comprehensive therapeutic effect on NAFLD by regulating the relevant targets and signaling pathways and also profoundly embodies the innovative concept of “treatment based on pattern differentiation” of TCM, which expands the scope of application of TCM and promotes the modernization of TCM, which is more in line with the development trend of traditional medicine in the future. Nowadays, with the innovative development of network pharmacology, bioinformatics, and computer technology, the combination of TCM and computational systems is getting closer and closer. From another perspective, network pharmacology techniques such as GO analysis and KEGG analysis have great similarity to the neuro-endocrine-immune (NEI) network, which was first proposed in 1977. The NEI network refers to the three major systems of nerves, endocrine, and immune systems that are independent and interact with each other, thus building a holistic network structure that maintains its equilibrium while at the same time promoting the coordinated development of multiple systems in the human body (Li, et al., 2014). These computational systems and related networks are proposed from the local concept to the overall concept of major progress and development, indicating that modern medicine focuses on the microscopic at the same time began to pay attention to the overall regulation of the research so that the connection between Chinese medicine and modern medicine more closely. It is believed that in the future, with the continuous development of experimental methods and computer technology, the active components, target, mechanism of action, compatibility law, and pharmacodynamic substance basis of TCM treatment of NAFLD will be more clearly described.

To this day, however, there are still challenges and problems in the application of network pharmacology due to several factors. First of all, the absorption and metabolism effects of oral Chinese medicines vary more significantly, and there is a considerable degree of potential research risk of false positives and false negatives, so in the future, we should focus on the medicine’s absorption, distribution, metabolism and excretion, and further screen the oral bioavailability of medicines to improve the accuracy of the study. Secondly, the mechanism of action between components and targets is still not completely clear. The components of TCM are very complex, and there are interactions between multiple components, making it difficult to clarify their mechanisms of action, which ultimately results in inaccurate prediction results (Hong, et al., 2017c). This requires us to develop databases with TCM characteristics or conduct relevant mass spectrometry analyses to further summarize and screen the effective active compounds of TCM, and to clarify the interconnections between compounds, targets, and diseases. Finally, a large number of network pharmacology studies are still in the prediction stage, and there are still many deficiencies in cellular or animal experiments, such as repeated experimental operations or more superficial studies, etc. In the future, we should carry out an in-depth excavation of the mechanism of action of Chinese medicines for the treatment of diseases and conduct more in-depth clinical or experimental studies.

Therefore, the ability to better apply network pharmacology techniques is of far-reaching significance for the prevention and treatment of relevant diseases in TCM and is of particular importance in future research. In summary, we believe that the use of network pharmacology is one of the most important ways to develop innovative medicines in TCM. With the continuous development and improvement of science and technology and the interpenetration and integration of network pharmacology and other disciplines, we believe that breakthroughs will be achieved in the prevention and treatment of NAFLD in TCM and the cause of TCM will have a more long-term development.
