# Supplementary material for: Chinese medicine in the treatment of non-alcoholic fatty liver disease based on network pharmacology: a review
Source: Front Pharmacol. 2024 Apr 17;15:1381712. doi: 10.3389/fphar.2024.1381712 (PMC11061375; doi:10.3389/fphar.2024.1381712)
Supplement: Supplementary file 1 [file Table1.pdf]

Supplementary TABLE 1 | Network pharmacology analysis of TCM in the treatment of NAFLD.

Reference	Formulae & Herbs	Objects	Main Compounds	Main Target	Main Signaling Pathway	Outcome
Li et al. (2021)	Andropanthes	Molecular docking	Andrographolide	IL1B, MAPK8, AKT1	TNF, IL-17, Toll-like receptor	Therapeutic effects through antioxidant, anti-inflammatory and modulation of new fatty acid synthesis
Wei et al. (2022)	Black ginseng	C57BL/6 mice, Molecular docking	Ginsenosides	STAT3, PIK3CA, VEGFA	Pathways in cancer, HIF-1	Black ginseng and its key components significantly improve liver histological changes in NAFLD mice
Hong et al. (2019)	Grapefruit peel	C57BL/6 mice	Narirutin, Neohesperidin, Naringin	VEGF-C, COL4A1	Toll-like receptor, P53, MAPK	Grapefruit peel may exert therapeutic effects on NAFLD via VEGF-C and COL4A1
Sun et al. (2022)	Lonicera japonica flos	HepG2 Cells, Molecular docking	Isochlorogenic acid B, Neochlorogenicacid, Quercetin-O-glucoside	CASP3,TNF, PTGS2	PI3K-AKT-mTOR, TNF, IL6-JAK-STAT3	Lonicera japonica Flos significantly down-regulates CASP3 and TNF-α expression in a NAFLD cell model
Luo et al. (2021)	Paeoniae radix alba	Molecular docking, Surface plasmon resonance	Catechin, Gallic acid	PRKAG1, PRKAG2, PRKAG3	Adipocytokine, Estrogen, HIF-1	Paeoniae Radix Alba is able to mitigate NAFLD through multiple metabolic pathways

Gong et al. (2022)	Eucommia ulmoides	HepG2 cells, Molecular docking	Eucommia ulmoides leaves 50	ALB, AKT1, TP53	NAFLD, Pathways in cancer, TNF	Key components of Eucommia ulmoides leaves significantly reduce lipid accumulation in the body and increase PPARγ expression
Wang et al. (2022)	Gynostemma pentaphyllum	Molecular docking	Quercetin, Rhamnazin, 3-methyleriodictyol	AKT1, GSK3B	PI3K-Akt	Gynostemma pentaphyllum inhibits insulin resistance by modulating the P13K-AKT signaling pathway
Mao et al. (2023)	Ganoderma lucidum	C57BL/6 mice	Resinacein S	NR1H4, CYP17A1, MMP1	T-cell receptor, TNF, MAPK	Resinacein S significantly improves lipid metabolism in hepatocytes, protects against steatosis, and attenuates liver injury
Li et al. (2023)	Schisandra chinensis	HepG2 cells, C57BL/6J mice	S. chinensis fruit ethanol, Lignans, schisandrol A	PIK3CA, GSK3β, JNK1	Pathways in cancer, TNF, AMPK	The key ingredient lignans modulates the insulin resistance signaling pathway, leading to therapeutic effects on NALFD
Xu et al. (2021)	Saffron	C57BL/6J mice	Crocetin	AKT1, MAPK1, STAT3	HIF-1, TNF, NF-kB	Crocetin modulates signaling pathways such as oxidative stress and diabetes to exert therapeutic effects in NAFLD
Hong et al. (2017b)	Radix salviae miltiorrhizae	C57BL/6J mice	Danshensu, Salvianolic acid b, Tanshinone iia	PPARα, MMP2, CYP1A2	None	Key components of Radix salviae miltiorrhizae regulate lipid metabolism in the body, thereby exerting a modulatory effect on disease

Wu et al. (2021)	Paederia scandens	Ross 305 chicks	Quercetin, Kaempferol	BCL2, TP53, SPP1	Toll-like receptor, Salmonella infection, Apoptosis	Paederia scandens significantly down-regulates serum triglyceride levels in NAFLD chickens
Chen et al. (2021)	Polygoni orientalis fructus	Human hepatocytes LO_2 cells	Syringic acid, Gallic acid, Protocatechuic acid	PPARG, IL6, TNF	AMPK, NF-kB	Polygoni Orientalis Fructus is effective in alleviating hepatic steatosis, oxidative stress, mitochondrial dysfunction and inflammatory response
Sun et al. (2022)	Artichoke	HepG2 cells, Molecular docking	Cynarine	TP53, ELANE, MMP9	PI3K-Akt, MAPK	Cynarine significantly reduces the fat deposition capacity of NAFLD model cells
Huang et al. (2023)	Cassiae semen	HepG2 cells, Molecular docking	CS ethanol extract	PIK3CA, CASP3,A 4	MAPK	Cassiae semen reduces EGFR and CASP3 expression by modulating the MAPK signaling pathway and ultimately reduces intracellular lipid accumulation
Wang et al. (2021a)	Blueberry	HepG2 cells,	Quercetin, Kaempferol, Catechin	AKT1, IL6, VEGFA	TNF, Pathways in cancer, NF-kB	Blueberry leaves can exert positive effects on NAFLD by modulating relevant targets and apoptotic pathways
Jiang et al. (2022)	Silybum marianum	C57BL/6J mice	Arachidonic acid, (-)-taxifolin, Stigmasterol	IL-6, MAPK1, AKT1	Pathways in cancer, Hepatitis B, HIF-1	Silybum marianum is able to down-regulate MAPK1, IL-6 and other proteins and increase AKT1 protein expression to exert therapeutic effects

Zhang et al. (2022)	Huanglian-Wuzhuyu herb pair	C57BL/6 mice, Molecular docking	Berberine, Epiberberine, Coptisine	NLRP3, ASC, Caspase-1	Metabolism, PPAR, AMPK	Huanglian-Wuzhuyu herb pair inhibits NLRP3 inflammasome and reduces hepatic steatosis and inflammation
Nie et al. (2020)	Chaihu Shugan powder	Wistar rats, Molecular docking	Naringin, Isorhamnetin, Kaempferol	FXR, PPARα,PPARγ	Tumours or cancers, Hepatitis B, Th17 cell differentiation	Chaihu Shugan powder was able to improve liver histopathology, liver lipids and body weight in a rat model
Wei et al. (2021)	Sinisan	Wistar rats	Quercetin, Mairin, Jaranol	STAT3,CTNNB1, MAPK1	Jak2/STAT3	Sinisan reduces hepatic steatosis and hepatic inflammation and inhibits hepatocyte apoptosis
Li et al. (2019b)	ShengMai-Yin and Ganmaidazao decoction combination	C57BL/6J mice	Quercetin, Kaempferol, Ursolic acid	INSR, PPARG, TNF	PI3K-Akt, AMPK, PPAR	ShengMai-Yin and Ganmaidazao decoction combination is able to reduce the size of adipocytes and decrease lipid deposition in the liver
He et al. (2022)	Shenqi pill	SD rats	Quercetin	PTGS2, JUN, MYC	IL-17, Human cytomegalovirus infection, TNF	Shenqi Pill significantly improves hepatic inflammation, lipid metabolism and liver injury in NASH rats
Liu et al. (2021)	Erchen decoction	C57BL/6J mice	Quercetin, Beta-sitosterol, Sitosterol	HMGCR, HTR2A, MMP2	TNF, PI3K-Akt, Toll-like receptor	Erchen decoction is able to reverse the levels of inflammatory factors in the body, thereby inhibiting the exacerbation of liver inflammation

Huang et al. (2020)	Erzhi pill	Molecular docking	Luteolin, Linarin	SRC, PIK3R1, AKT1	Pathways in cancer, PI3K-Akt, FoxO	Erzhi pill exerts preventive effects on chronic liver disease through a multi-component, multi-target, multi-pathway approach
Yang et al. (2020)	Fuzi Lizhong decoction	Wistar rats	Codonopsine, (S)-6-Gingerol, Delcosine	TP53, JUN, DPP4	p53, PPARG	Fuzi Lizhong decoction regulates the body's immune system by modulating relevant signaling pathways, ultimately improving NAFLD
Gao et al. (2022)	Ganlu powder	Molecular docking	Berberine, Epiberberine, Jatrorrhizine	AKT1, TP53, MAPK8	TNF, HIF-1, Pathways in cancer	Ganlu powder is able to modulate the TNF signaling pathway by affecting AKT1 to achieve therapeutic effects
Hu et al. (2020)	Xiaochaihu decoction	None	Quercetin, Kaempferol, Stigmasterol	AKT1, IL6, JUN	AGE-RAGE, HIF-1, IL-17	Xiaochaihu decoction exerts positive therapeutic effects on NAFLD by regulating the body's oxidative stress, metabolism and immunity
Shi et al. (2022)	San-Huang-Tang	C57BL/6J mice, HepG2 cells	Palmidin C, Rheidin B, Sennidin C	CP2E1, P53, HMOX1	INSR/IRS1/AKT/FoxO1	San-Huang-Tang significantly ameliorates insulin resistance in NAFLD mice
Wu et al. (2021)	ZeXie decoction	SD rats	Alisol C Monoacetate, Alisol C, Alisol B 23-Acetate	HMGCR, SREBP-2, MAPK1	Pathways in cancer, PPAR, AGE-RAGE	ZeXie decoction exerts therapeutic effects on NAFLD by modulating NF-κB p65, HMGCR, and MAPK1

Zhang et al. (2020a)	Chaihu Lizhong Tang	Molecular docking	α-Amyrin, 8B-Ethoxy Atractylenolide III, Beta-Sitosterol/Sitosterol	AKT1, ALB, IL6	AGE-RAGE, IL-17	Chaihu Lizhong Tang has obvious therapeutic effects against NAFLD and is characterized by the synergistic effects of multi-component, multi-target and multi-pathway
Wang et al. (2020b)	Jiangzhi decoction	Human hepatic L02 cell line, Molecular docking	Hyperin, Emodin, Emodinanthrone	TNF, IGF1, IL1B	Nuclear receptor transcription, Glutathione conjugation, Interleukin-4 and 13	Jiangzhi decoction modulates key molecules associated with nuclear receptor transcription and lipid metabolism to alleviate hepatocellular steatosis
Gao et al. (2020)[87]	Qushi Huayu decoction	None	Quercetin, Luteolin, Isorhamnetin	ATK1, JUN, ALB	Pathways in cancer, PI3K-Akt, PPAR	Qushi Huayu Decoction exerts hepatoprotective effects by modulating the cancer pathway, P13K-AKT signaling pathway and PPAR signaling pathway
Wu et al. (2023)	Qutan Huoxue decoction	SD rats, Primary hepatocytes	Wogonin, Kaempferol, Isorhamnetin	MAPK14, AKT1, FOX	SCOS1/NF-κB/TLR4	Qutan Huoxue decoction exerts hepatoprotective effects by activating SCOS1/NF-κB/TLR4 signaling pathway
Xu et al. (2022)	Shen-Shi-Jiang-Zhuo formula	SD rats	Ellagic acid, Mandenol, Mollugin	PTGS2, RXRA, NCOA2	PI3K-Akt	Shen-Shi-Jiang-Zhuo Formula ameliorates hepatic lipid accumulation and fibrosis, and reduces collagen deposition in NAFLD rats
Yang et al. (2022b)	Shugan Xiaozhi decoction	Molecular docking, Molecular dynamics simulation	Gallic acid, Chlorogenic acid, Isochlorogenic acid A	RELA, IL-6, VEGFA	PI3K-Akt, MAPK, Insulin resistance	Shugan Xiaozhi decoction is able to modulate the active substances and key targets in multiple related pathways to exert therapeutic effects

Yin et al. (2022)[91]	Shuangyu Tiaozhi decoction	Rats, HepG2 cells	Diosgenin, Hancinol, Denudatin B	ESR1, FASN, mTOR	Thyroid hormone, Insulin resistance, HIF-1	Shuangyu Tiaozhi decoction ameliorates lipid deposition, inflammation and insulin resistance and exerts anti-NAFLD effects
Wang et al. (2021c)	Fufang Zhenzhu Tiaozhi capsule	Minipigs	Quercetin, Kaempferol, Luteolin	STAT3,AKT1, JUN	PI3K-AKT, HIF-1α	Fufang Zhenzhu Tiaozhi capsule can increase the phosphorylation of PI3K-AKT and decrease the expression of HIF-1α, and ultimately improve the liver metabolism
Zhi et al. (2023)	GanShuang granules	SD rats, Molecular docking	Resveratrol, Naringin	AKT1, CASP9,TNF	Oxidative stress, NF-κB, Apoptosis	GanShuang granules ameliorate the inflammatory state and restore the abnormal lipid accumulation in the liver of NAFLD rats
Li et al. (2018)	Jian-Gan-Bao	C57BL/6J mice	Tanshiquinone B, Miltirone, Miltirone I	IL6, TNF, SREBF1	Inflammation, Fatty acid oxidation, TNF production	Jian-Gan-Bao has significant preventive effects on acute and chronic alcoholic liver disease and NAFLD
Zhou et al. (2021)	Jiangzhi granule	C57BL/6 mice	Emodin, Resveratrol, Quercetin	TNF, IL6, CCL2	IL-17, TNF, Toll-like receptor	Jiangzhi Granule lowers liver cholesterol levels and also reduces inflammatory factors in the liver
Zhang et al. (2020b)	Kangtaizhi granule	SD rats, HepG2 cells	Quercetin, Linoleic acid, Kaempferol	PPARG, ADIPOQ, AKT1	AMPK/mTOR	Kangtaizhi granule ameliorates hepatic steatosis and lipid accumulation in rat and HepG2 cells by acting on the AMPK/mTOR signaling pathway

Jia et al. (2022)	Mailuoning oral liquid	C57BL/6 mice, HepaRG cell line	Neochlorogenic acid, Chlorogenic acid, Cryptochlorogenic acid	AKT1, MAPK1, MAPK3	PGC-1α-PPARα	Mailuoning oral liquid exerts hepatoprotective effects by modulating the PGC-1α-PPARα signaling pathway
Tan et al. (2023)	Yinzhihuang granule	C57BL/6 mice, Molecular docking	Apigenin 7-glucoside, Apigenin 7-O-glucuronide, Baicalein	AKT1, MMP9, EGFR	Sphingolipid metabolism, Glycerophospholipid metabolism, Caffeine metabolism	Yinzhihuang granule significantly improves hepatic lipid metabolism and enhances intestinal barrier function
